# Genetic Diversity Evaluation and Conservation of Kam Fragrant Glutinous Rice (*Oryza sativa* L.) Germplasm in Southeast Guizhou, China

**DOI:** 10.3390/plants10091898

**Published:** 2021-09-14

**Authors:** Qi-Yi Lei, Jiang-Ju Zhou, Yong Xiong, Wen-Hua Zhang, Jing Luo, Chun-Lin Long

**Affiliations:** 1 College of Life and Environmental Sciences, Minzu University of China, Beijing 100081, China; leiqiyi@126.com (Q.-Y.L.); xiongyong185@sohu.com (Y.X.); 2 College of Life and Environmental Sciences, Kaili University, Guizhou 556000, China; kxky2012@126.com (J.-J.Z.); zhwh0808@sina.com (W.-H.Z.); klluojing@126.com (J.L.); 3 Kunming Institute of Botany, Chinese Academy of Sciences, Kunming 650201, China

**Keywords:** rice varieties, landraces protection, morphological traits, genomics, traditional folk classification

## Abstract

The genetic diversity of rice germplasm is the basis for increases in rice yield and quality. The collection, assessment, and protection of the genetic diversity of rice germplasm is important for achieving sustainable agriculture and assuring food security. Many underdeveloped indigenous areas have abundant and valuable rice germplasm resources. However, in-depth assessments of the genetic diversity of rice germplasm from these areas and studies related to protecting these traditional cultures are not available. In this study, from 2005 to 2016, the authors have conducted in-depth evaluation of the genetic diversity of Kam fragrant glutinous rice germplasm resources in southeast Guizhou by using multidisciplinary comprehensive methods such as ethnobotany, cultural anthropology, and modern molecular markers. In total, 376 Kam fragrant glutinous rice samples from 42 villages in the Dong community in southeast Guizhou were collected. Agronomic traits of panicles were complex and exhibited diversity. Some varieties had good disease resistance and adaptation to cold and wet climates. The Dong people named the Kam fragrant glutinous rice varieties by using seven elements, including diverse traits, growth environment, and origin. Traditional folk classification, in addition to morphology and biological analysis using molecular markers, indicates that Kam fragrant glutinous rice includes 91 varieties. Kam fragrant glutinous rice comprises a very high number of varieties, most of which are *japonica*-type and exhibit a high level of genetic diversity. The traditional folk classification of Kam fragrant glutinous rice by the Dong community is consistent with the biological classification. The traditional naming of Kam fragrant glutinous rice provides an important reference for understanding its genetic diversity. The high level of genetic diversity in Kam fragrant glutinous rice is not only related to the natural environment of the area but also tightly linked with the abundant and diverse Dong ethnic traditional cultures, which has led to protection of Kam fragrant glutinous rice’s genetic diversity.

## 1. Introduction

Rice (*Oryza sativa* L.) is cultivated in over 100 countries and provides food for millions of people [1,2]. Thus, rice is considered one of the most important crops in the world [3]. Studies have shown that abundant rice landraces are preserved in a few underdeveloped ethnic and remote areas in developing countries, such as the southwest region of China [4,5], the northeast region of India [6], Northern Thailand [7], Northern Indonesia [8], Nepal [9], and Niger [10]. The areas mentioned above have extremely abundant rice germplasm resources that play important roles in maintaining local agricultural biodiversity, preventing diseases and pests, increasing yield, and providing nutrition [11,12]. Rice genetic diversity is the core of agricultural biodiversity and an important resource for increasing rice yield and quality [13]. The collection, assessment, and protection of the diversity of rice germplasm resources form a crucial foundation for sustainable agriculture and food safety. Areas in Southeast Asia have experienced a significant loss in rice diversity due to economic and political globalization, the adoption of high-quality, high-yield rice varieties from modern agriculture, and changes in traditional farming systems and ethnic cultures. Agricultural biodiversity is suffering from unprecedented destruction [14]. This trend is becoming more severe, and food and agricultural ecology are under serious threat; thus, these issues have gained worldwide attention. As a result, the assessment and protection of the genetic diversity of rice germplasm resources have become trending research topics in recent years.

China possesses abundant resources of glutinous rice varieties and has a long history of rice cultivation. During its long developmental process, glutinous rice gradually formed ecotypes suitable for cultivation in different regions. According to the statistics in the *Catalog of Rice Germplasm Resources in China* (1988–1993) on the glutinous rice varieties in the national crop germplasm bank, there is a total of 9107 varieties (7094 *japonica*-type and 2013 *indica*-type), including duplicates from each province. Among them, there were 2204 varieties in Guangxi, 1770 in Guizhou, and 1461 in Yunnan [15]. The glutinous rice variety resources from these three provinces are extremely abundant, comprising nearly half of the glutinous rice varieties in the entire country. There are several reports on the glutinous rice germplasm resources in Yunnan and Guangxi, China, as well as assessments of their diversity [16,17,18,19]. However, there are few studies on the abundant glutinous rice germplasm resources of Guizhou Province. Surprisingly, the southeast and south areas of Guizhou Province, where glutinous rice was consumed historically as a staple, are not listed as glutinous rice cultivation regions [20] or glutinous rice cultural districts [21]. Until now, the ethnic-minority areas in southwest China remain an abundant and diverse region of glutinous rice cultivation. The Dong community, located in the southeast Guizhou, has the largest number of glutinous rice varieties with a wide and concentrated cultivation. Cooked glutinous rice from these areas has earned the region the saying “the aroma of the rice being cooked in one home can be smelled by a hundred families living nearby.” As a result, the Dong people have called it Kam sweet rice (the Dong people refer themselves as Kam) [22], or Oux or Kgoux in the Dong language. These rice varieties are strongly adapted to the cold and wet climate in the high mountains and the poor soil. They have good resistance to pests and are included as “Specialty Rices” by the International Rice Commission of the Food and Agriculture Organization of the United Nations [23]. Moreover, the “rice–fish–duck” system centered with Kam fragrant glutinous rice in the Dong community in Congjiang County was listed in the Globally Important Agricultural Heritage Systems (GIAHS) in 2013. The Jiabang rice terrace in Congjiang County, which is mainly used for Kam fragrant glutinous rice cultivation, has become an important agricultural and cultural landscape. Currently, research on Kam fragrant glutinous rice includes preliminary studies in cultural anthropology, quality traits, and genetic diversity [24,25,26,27]. The collection and identification of the germplasm resources and the assessment of genetic diversity in Kam fragrant glutinous rice remain unexplored.

Due to political, economic, and cultural globalization, and especially the recent urbanization in China and the rapid development of the Ecological Immigrant Project in western China, a massive number of indigenous Miao and Dong people from the southeast Guizhou have migrated to the urban areas. This migration had a great impact on traditional ethnic and farming cultures. Additionally, the wide adoption of hybrid rice and highly effective cash crops have replaced traditional rice farming. The glutinous rice varieties in the southeast region of Guizhou Province decreased from 378 in 1983 to fewer than 100 in 2016 [28]. Since the beginning of the 21st century, diverse and high-quality Kam fragrant glutinous rice varieties have decreased dramatically. There is an urgent need to collect, rescue, and protect these precious local rice variety resources to assess the genetic diversity of Kam fragrant glutinous rice. The purpose of this study was to identify the current status of Kam fragrant glutinous rice varietal resources in the southeast region of Guizhou Province using multidisciplinary methods in ethnobotany and cultural anthropology, which included traditional naming, varietal type, quantity, characteristics, disease resistance, and ethnic traditional cultures. We also utilized modern molecular-marker labeling to analyze and assess the traditional classification and genetic relationships between the Kam fragrant glutinous rice varieties. We analyzed the causes of genetic diversity and future challenges. Our study aimed to provide important information for the improvement and utilization of local rice varieties and to serve as a reference to develop effective protection measures.

## 2. Results

### 2.1. Varieties, Main Characteristics, and Traditional Naming of Kam Fragrant Glutinous Rice

**Figure 1:**
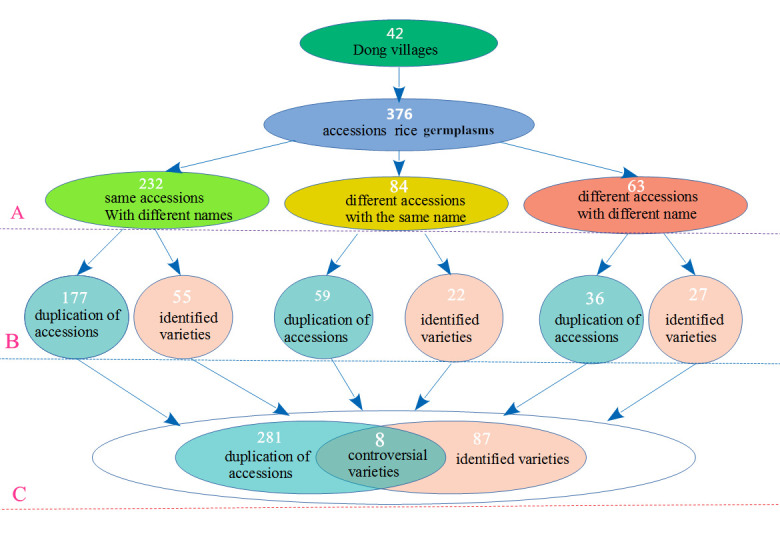
Kam fragrant glutinous rice varieties identified by folk classification at three levels. (**A**) The varieties planted by the ordinary farmers were identified. (**B**) The village varieties were identified by the elders, the patriarch, or the leader of a village. (**C**) Representatives composed of elders, patriarchs, and village leaders from 13 villages with large populations and a variety of resources were selected to compare and identify all the germplasm. Finally, those germplasms with different names but of the same variety were merged, different varieties with the same names were separated, 281 were identified as duplicate varieties, and 95 Kam fragrant glutinous rice varieties were identified, including 8 controversial varieties.

**Figure 2:**
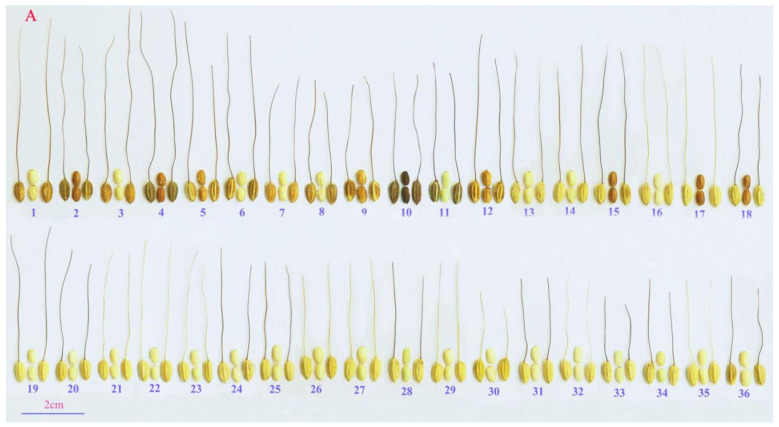

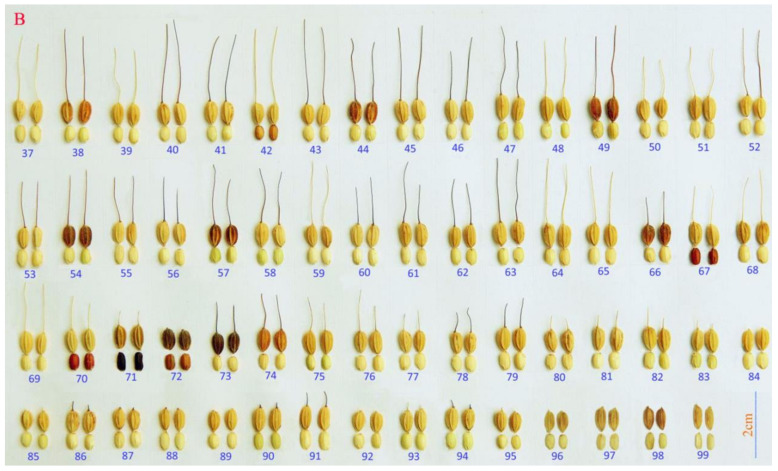
(**A**) The diversity of morphological traits of panicles in Kam fragrant glutinous rice. (**B**) The diversity of morphological traits of spikelets and seeds in Kam fragrant glutinous rice. No. 96–99 are the control varieties, in which 97 and 98 are glutinous *japonica*, 96 and 99 are non-glutinous *indica*.

From the 42 main Dong villages in the study area, 376 Kam fragrant glutinous rice germplasms were collected. Villagers were organized at three levels and identified the rice germplasm according to the Dong ethnic traditional classification (Figure 1). Different varieties with the same names and varieties with the same names that were different were both very common. After separating the different varieties with the same names and combining the same varieties with different names, we identified 95 local Kam fragrant glutinous rice varieties (including 8 controversial varieties) (Figure 1). The seeds of Kam fragrant glutinous rice often have a long awn, and differences in awn color, glume color, and grain color were abundant. Among the 95 Kam fragrant glutinous rice varieties in this study, 90.53% were varieties with awn (Figure 2A,B). The number of varieties with awns 3–6 cm in length was 61.05%. The awn colors were primarily yellow (34.74%) and brown (23.16%), followed by black (14.74%) and red (7.37%). Varieties that had a yellow glume color were the most abundant (73.68%) group, followed by the varieties with brown glumes (8.42%). For the grain color, ivory-white-colored varieties were the most common group (69.74%), followed by those with light-green (15.79%) and brownish-red ones (4.21%) grains.

**Figure 3:**
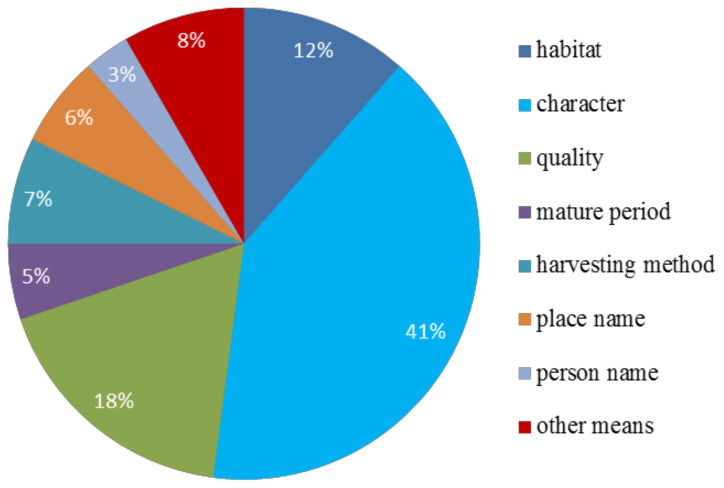
The seven naming methods for Kam fragrant glutinous rice.

The most distinct morphological traits in Kam fragrant glutinous rice included awn shape, awn color, glume color, and grain color. These traits formed the primary basis for the classification of Kam fragrant glutinous rice germplasm by the Dong people (the varieties’ codes and Dong ethnic names are shown in Supplementary Material Table S1). Traditional naming of Kam fragrant glutinous rice includes three parts (basic name + origin name + description). Among the 95 Kam fragrant glutinous rice varieties in this study, there were 7 naming methods base on character traits, quality, growth environment, locations, maturity stage, person name, and other information. Refer to Figure 3 for specific information.

### 2.2. Total Genetic Diversity

Using the 20 selected SSR markers, we performed fluorescence-labeled SSR genotyping using capillary electrophoresis on 95 Kam fragrant glutinous rice varieties from six representative Dong villages and four controls varieties. The assessment of genetic diversity is shown in Table 1. Using 20 microsatellite markers on 99 rice varieties, a total of 128 alleles were detected, and the number of alleles varied from 2 to 17, with an average of 6.4. The percentage of polymorphic bands (PPL) was 100%; the average polymorphism information content (PIC) values varied from 0.2814 (RM164) to 0.7751 (RM219, RM247), with an average of 0.5576. Nei’s genetic diversity index (H) exhibited an average value of 0.6061 and varied from 0.2972 (RM164) to 0.8000 (RM219). The Shannon diversity index (I) had an average value of 1.2142, and the average heterozygosity (Ave_Het) was 0.0092. The average gene flow parameter Nm was 0.0038. Different indices showed that the SSR primers had many differences, indicating high genetic diversity. Among them, the most effective SSR primer was RM229, with a Shannon diversity index above 1.5, 8 alleles, and effective genes greater than 4.0.

### 2.3. Genetic Distance

**Figure 4:**
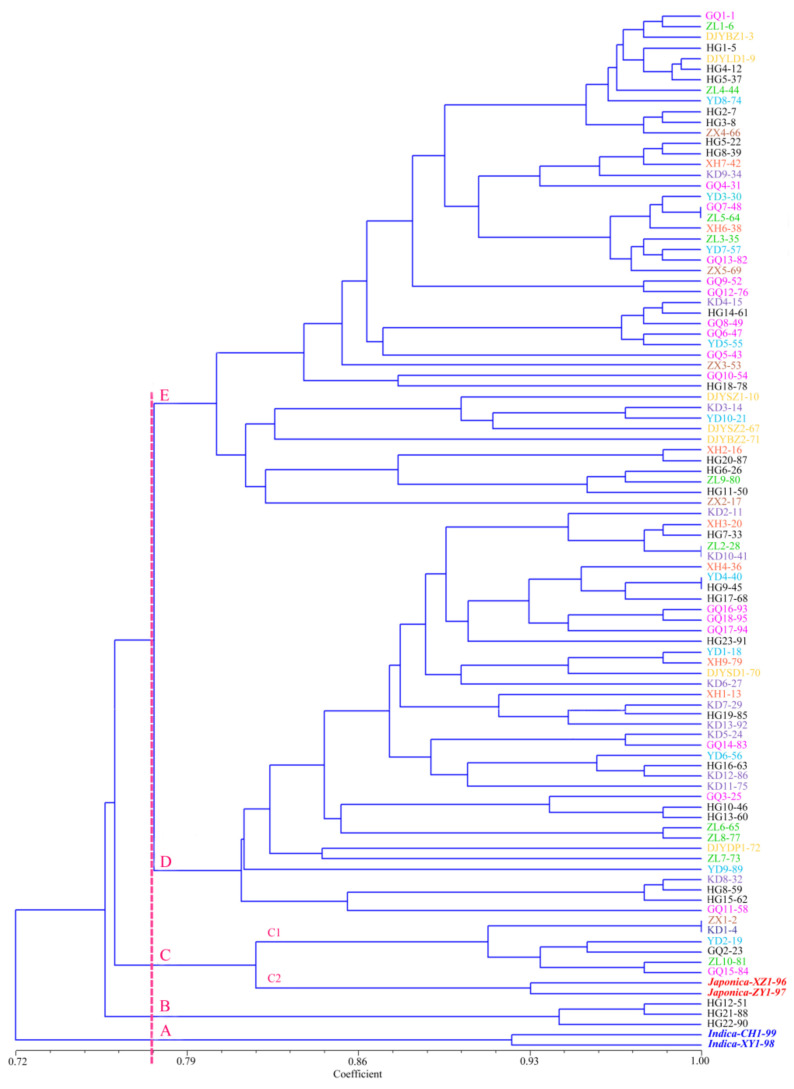
Cluster dendrogram of 99 rice landrace based on genetic similarity. No. 96–99 are the control varieties, in which ZY1-97 and XY1-98 are glutinous *japonica*, XZ1-96 and CH1-99 are non-glutinous *indica*.

UPGMA clustering was based on comparisons of genetic similarity coefficients among varieties (Figure 4). The genetic similarity coefficients among the 95 Kam fragrant glutinous rice varieties varied from 0.72–1.00. When the coefficient was 0.7513, the 95 Kam fragrant glutinous rice varieties and four control varieties were grouped into four clusters (A, B, C, and D). Among them, cluster A included 2 Indica-type control varieties (glutinous XZ1-98 and non-glutinous CH1-99). Cluster B included 3 Indica-type Kam fragrant glutinous rice varieties (HG12-51, HG21-88, and HG22-90) from Huanggang Village. Cluster C included C1 and C2, two subclusters. C1 included 2 *japonica*-type control varieties (non-glutinous XY1-96 and glutinous ZY1-97). C2 included 6 glutinous Kam fragrant glutinous rice varieties. Cluster D was the largest and was divided into subclusters D1 and D2, with a total of 86 Kam fragrant glutinous rice varieties. Cluster D was the main body of the cluster figure. Among the 95 varieties shown in Figure 1, except for the yellow-glume varieties in the 22 samples with special glume colors, 19 were grouped in subcluster D2. The other 5 samples, Nos. 2, 4, 11, 72, and 73, were distributed in other clusters. Almost all the special glume color varieties were clustered within one subcluster.

### 2.4. Principal Coordinate Analysis (PCoA) of All Varieties Tested

**Figure 5:**
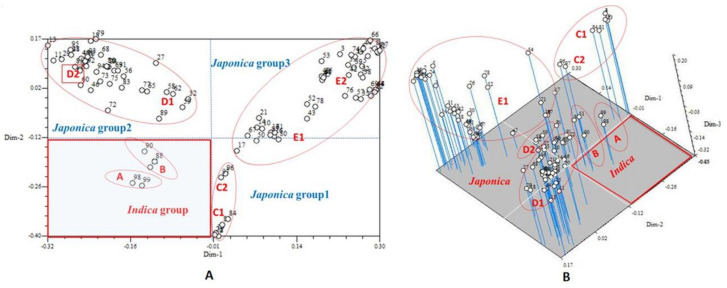
The 2D (**A**) and 3D (**B**) PCoA analysis of 99 rice samples based on 20 pairs of SSR primers.

This study used NTSYS-pc 2.10e to conduct PCoA analysis on all 99 germplasm resources tested. A 3D PCoA clustering plot was obtained (Figure 5B). Along the 3 axes, all tested varieties were clearly grouped into five clusters: clusters A and B, C, D, and E were concentrated in zones 1, 2, 3, and 4, respectively. Cluster A included two Indica-type control varieties (Nos. 98 and 99). Cluster B included three Kam fragrant glutinous rice varieties from Huanggang Village (Nos. 51, 88, and 90) and was distant from cluster A, but it appeared to cluster with group C in the 3D plot. This cluster might be a transitional cluster between Indica and *japonica* rice. Cluster C included two *japonica*-type control varieties and six Kam fragrant glutinous rice varieties. Variety No. 17 was adjacent to this cluster. Clusters D and E were more concentrated. In addition, the results from a 2D PCoA clustering (Figure 5A) were consistent with the results mentioned above. The clustering of rice varieties obtained from the 2D and 3D PCoA plots and UPGMA clustering graph were highly consistent.

### 2.5. Analysis of the Genetic Diversity of the Community of the Different Community of Kam Fragrant Glutinous Rice

Eight representative communities, which contained 95 folk varieties, were selected to test their genetic diversity. As is shown in Table 2, the results indicated that the percentage of polymorphic loci in each community was more than 95%. The order of Nei gene diversity index, from high to low, was Huanggang (0.6149), Gaoqian (0.5677), Yandong (0.5646), Zhanli (0.5420), Dongjiayuan (0.5505), Xiaohuang (0.4929), kengdong (0.4667), and Zhaoxing (0.4626). The diversity Nei’s gene, the Shannon index, and the percentage of polymorphic all indicated that the genetic diversity of each community (i.e., each Dong Village) of Kam fragrant glutinous rice was high and the genetic background varies greatly.

The genetic distance of eight main Dong villages is between 0.0406 and 0.2457 (Table 3). Dongjiayuan and Kengdong had the longest genetic distance, while Gaoqian and Yandong had the shortest genetic distance. The genetic similarity coefficient ranged from 0.7822 to 0.9602. Gaoqian and Yandong had the greatest genetic similarity, while Dongjiayuan and Kengdong had the smallest genetic similarity.

**Figure 6:**
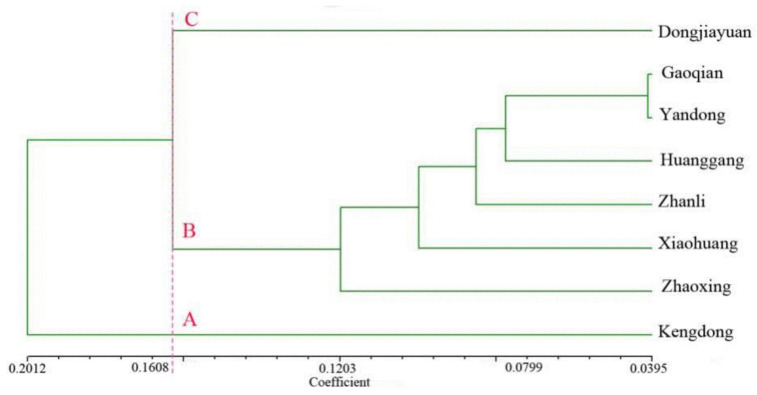
The genetic distance of different varities of Kam fragrant glutinous rice from eight Dong villages.

Based on the genetic diversity analysis of Kam fragrant glutinous rice population in southeastern Guizhou, the genetic distance maps of representative kam fragrant glutinous rice varieties from eight different Dong villages were obtained by UPGMA clustering method (Figure 6). The varieties from eight Dong villages were divided into three branches (A, B, C). Branch A and C only include one village, Kengdong and Dongjiayuan, respectively, and they have the farthest genetic distance. Branch B is the basis of the whole genetic clustering map, including six Dong villages, in which Gaoqian and Yandong have the closest genetic distance. According to the cluster map of genetic distance, most villages’ genetic distance do not correspond to the actual geographical location, and there is no correlation between genetic distance and geographical location.

## 3. Discussion

### 3.1. The Traditional Naming and Classification of Kam Fragrant Glutinous Rice Provides an Important Foundation for Understanding Its Genetic Diversity

The Kam fragrant glutinous rice varieties display phenotypic diversity and traits, including awn shape, awn color, glume color, and grain color, that are abundant and diverse. The Dong people named and classified these Kam fragrant glutinous rice varieties based on their qualities, growth environments, names of origin, maturity stages, and harvesting methods. The Kam fragrant glutinous rice variety type is derived from three important factors: the name, origin, and description. Sociologists and ethnologists Durkheim and Mauss [29] once considered primitive classification as the origin of human cognition and its universal foundation. Naming and classification based on folk traditions have become important ways to understand and study the germplasm resources of Kam fragrant glutinous rice. American anthropologist Brush [30] pointed out that “Naming and categorizing types of crops are popular pastimes that open doors to many aspects of rural life, including knowledge of the environment, the relationship between humans and land, the hard livelihood of struggling with the earth, and ingenuity in adapting different technologies to a specific environment.” These views are in high agreement with our practice of investigating and confirming the Kam fragrant glutinous rice diversity of variety resources in this area. It shows that naming and classification based on folk tradition have become important ways to understand and study the germplasm resources of Kam fragrant glutinous rice.

Due to differences in accents among Dong villages, descriptions of varietal characteristics, trading, and the origins of rice varieties are different. Thus, there are major differences among varietal names, which explains why the same varieties have different names and different varieties have the same name. To confirm the variety of resources in the area, we conducted significant field studies over the past 10 years. We repeated our study of the 42 Dong villages at least once every three years and collected a total of 376 Kam fragrant glutinous rice germplasms from the villages. Using a multistrategy identification by representatives from individuals, families, and the Dong communities, we combined the same varieties with different names and separated different varieties with the same names. Finally, we concluded that a total of 95 Kam fragrant glutinous rice varieties were currently present in the area (including eight controversial varieties). Using SSR markers, our study showed that the genetic similarity value was 1 between eight controversial varieties: Nos. 2 and 4, Nos. 48 and 64, Nos. 28 and 41, and Nos. 40 and 45. In addition, their seeds had similar characteristics. Therefore, these eight controversial varieties should be combined into four varieties, as some villagers suggested. Currently, 91 Kam fragrant glutinous rice varieties exist in this area. We compared the traditional folk classification with modern molecular biological methods and found that the two classification methods yielded highly consistent results. The traditional folk classification provides important clues for the understanding of varietal diversity. Our study gave a positive answer to the question of “whether the existence of varietal naming is a symbol of the existence of diversity,” which was asked by the International Rice Research Institute (IRRI) scholar Sadiki [31], and further indicated that “studying varietal names should be an important entry point in studying crop genetic diversity and distribution patterns in the agroecosystem.” [32]. Thus, investigating the naming system of local rice varieties is important for understanding agricultural biodiversity.

### 3.2. Genetic Diversity of Markers

This study used 20 SSR primers to analyze the genetic diversity of 99 Kam glutinous rice varieties from the southeast region of Guizhou Province (including two *japonica*-type and two *indica*-type local varieties from Guizhou as controls). We detected an average of 6.4 alleles per locus, which was higher than that of local varieties currently cultivated in Yunnan Province (5.8 alleles per locus) [33], including 80 local rice varieties (4.37 alleles per locus) [34], and higher than that of 329 traditional rice cultivars in China (5.7 alleles per locus) [35]. We found a significantly greater number of alleles than those found in previous studies using 80 local rice varieties in Guizhou (4.381 alleles per locus) [26], 224 *japonica*-type local varieties in Tai Lake Basin (3.6 alleles per locus) [36], and 314 accessions rice germplasms from northern Laos (3.49 alleles per locus) [37]. However, the amount of allelic variation we detected was significantly lower than that (10.5 per locus) detected in 288 common wild and cultivated rice varieties in China [38] (Yu et al. 2004). The average PIC value of Kam glutinous rice was 0.5576, which was higher than the PIC value (0.1765) obtained from 1610 medicinal wild rice varieties in Guangxi using 25 SSR primers [39] and higher than the PIC value (0.36) of 62 conventional rice varieties from the Yangtze River basin analyzed by 50 SSR primers [40]. Our obtained PIC values were also higher than the average PIC value (0.47) obtained from rice in the Chillán region of Chile using 30 SSR primers (Becerra et al. 2017) and the PIC value (0.30) of 24 rice varieties from Iran using 36 SSR markers [41]. These data indicate that Kam glutinous rice in the Dong area of the southeast region of Guizhou Province has a high level of genetic diversity.

We performed PCoA analysis using NTSYS software on all 99 Kam glutinous rice accessions used in this study and obtained 2D and 3D PCoA plots that showed consistent clustering results using the UPGMA clustering method. Classification information obtained from three analyses had the following characteristics: (1) the clustering tree graph created using the UPGMA method provided abundant information when analyzing relationships among varieties; (2) the 2D PCoA plot provided a flat and direct view of the classification of glutinous rice, especially for the transitional populations or individuals, although the information was not comprehensive; and (3) the 3D clustering directly illustrated and provided detailed genetic information and data about the relationships among the glutinous rice varieties in different layers and directions. Combining the data from different analytical methods not only provides supplementary information and consistency but is also valuable for a comprehensive understanding of the genetic structure and classification status of glutinous rice. Through both the genetic similarity coefficient and the population structure analysis based on the PCoA, the glutinous rice varieties were divided into three groups: the japonicaclinous type (a large percentage), the indicaclinous type (a small percentage), and transitional individuals.

The results of genetic diversity analysis of eight representative communities showed that the genetic diversity of each community (that is, each Dong villlage) was high, the genetic background was different, the genetic distance of most communities did not completely correspond to the actual geographical location, and the genetic distance of the community had no correlation with the geographical location. These results have been rarely reported in previous studies. Although they are inhabited by Dong ethnic people the genetic diversity level of each village is high because of the great differences in farming environment, diet culture and custom in each village, and the minimal exchange of rice varieties in the past. However, in the past 20 years, with the rapid development of traffic in this area, variety exchange has been strengthened and the government has taken certain protection measures, the correlation between genetic distance and geographical location of the community is not obvious.

### 3.3. Formation and Protection of Kam Fragrant Glutinous Rice Diversity

In the agricultural system, rice diversity is affected by genetic population structure, natural selection from the surrounding environment, and human selection and management [4]. The southeast Guizhou is mountainous with rich water resources and a forest coverage reaches greater than 75%. The farming environment is complex and variable. Among the 95 Kam fragrant glutinous rice varieties in this study, 12 varieties originated from an altitude lower than 500 m, 33 from an altitude between 500 and 800 m, and 50 from an altitude higher than 800 m. Kgoux weenx ninl, Kgoux yangc longl, and other 9 varieties exhibited vigorous growth in the alpine region and in cold, wet environments. The Kgoux yangc longl and Kgoux gkaox dah laox varieties can reach maturity with only 2–4 h of sunlight every day. These varieties have long and hard awns to protect against insects. They are well adapted to an environment with short daylight, low air and water temperatures, and a shortage of resources caused by frequent early frosts and heavy fog. The two varieties Kgoux lieec jul and Kgoux bic ral are adapted to various environmental conditions and exhibit high yield, high quality, and extremely high resistance to rice blast and the rice stem maggot. The fast-maturing varieties Kgoux logc xil maenv and Kgoux jinc dongl require approximately 60 days from seedling transplant to maturity, exhibiting excellent adaptation to a short rainy season or a smaller area of farmland. This series of findings of the special resistance of Kam fragrant glutinous rice varieties provides very important clues for further digging into the utilization and protection of these important genetic resources.

Many studies have shown that traditional agricultural patterns and cultures of indigenous societies are important contributors to the preservation of crop germplasm diversity [5,6,25,28,31,35,42]. In long-term production practice, the Dong nation produced Kam fragrant glutinous rice by the traditional farming method of intercropping and rotation and made use of rice fields to breed fish and ducks, forming the original “rice–fish–duck” agricultural ecosystem. The system is not only beneficial for pest control and increasing the yield of Kam fragrant glutinous rice but also provides rich agricultural and sideline products, such as fish, ducks, and eggs. It has been a long-term scientific solution to the major livelihood problems of mountain nationalities. Therefore, the system has been designated a Globally Important Agricultural Heritage System (GIAHS). In recent years, with the protection and development of the GIAHS project, the agricultural cultural landscape based on Kam fragrant glutinous rice has been highlighted for its economic and ecological benefits, and the traditional farming knowledge has been passed on and developed. It can effectively promote the protection of the diversity of Kam fragrant glutinous rice germplasm resources, which shows that traditional farming culture plays a very important role in maintaining the genetic diversity of endangered crop germplasm resources.

The Dong people in the southeast region of Guizhou Province are an indigenous ethnic group whose members are diligent, kind, and talented singers and dancers and have diverse customs and manners. The Grand Songs of Dong are world-famous. The Dong ethnic and traditional cultures are rich and colorful. In our study, we found that the Dong people have more than 160 traditional holidays per year, and glutinous rice is almost always used at each festival. Glutinous rice is necessary for etiquette and daily activities, such as visiting family and friends, weddings, funerals, social interactions, and religious beliefs. Thus, every etiquette or custom in the daily life of the Dong people is tightly linked with glutinous rice. According to records, during the Ming and Qing dynasties, more than 10 ethnic groups living in the southeast region of Guizhou Province commonly grew glutinous rice and ate it as the only staple food. Later, three large-scale movements to “transform glutinous rice into nonglutinous rice” took place [28], and modern hybrid rice was highly promoted in the late 20th century. However, these events did not affect the traditions of the ethnic minorities, including the Miao and Dong, who consume glutinous rice as a staple food and widely plant Kam fragrant glutinous rice. Traditional glutinous rice-farming culture remains in the life of the Miao and Dong people in this area. Some Kam fragrant glutinous rice varieties, such as Kgoux naeml, Kgoux padh nyox, and Kgoux bic bagh, are not only used in rice-wine brewing and festivals but also possess important medicinal properties for treating tuberculosis and gynecological problems. Because of the excellent quality of Kam fragrant glutinous rice, including its fragrance and delicious taste, it is still widely used by the Dong people in social interactions, etiquettes and customs, medicine, and art. Some Dong villages still only eat glutinous rice. Glutinous rice is also an important spiritual food for the Dong people. To assure the continuation of their eating habits and to satisfy the needs of various cultural activities, the Dong people created a special production system and gained comprehensive knowledge of production practices. They selectively bred a series of Kam fragrant glutinous rice varieties suitable for the farming environment in each village and therefore protected the genetic diversity of Kam fragrant glutinous rice. It fully shows that traditional Dong traditional culture is the most important factor in Kam fragrant glutinous rice diversity and verifies that cultural diversity is closely related to biodiversity [43]. As a result, when developing measures for protecting the diversity of endangered crop species, such as Kam fragrant glutinous rice, including ex situ conservation, in situ conservation, or dynamic new protection modes for farmers, it is important to consider the relationship between crops and the environment, and, more importantly, the positive role of ethnic traditional culture in protecting the diversity of endangered crop species.

## 4. Conclusions

In summary, the Dong area in southeastern Guizhou maintains a very traditional culture of Kam fragrant glutinous rice cultivation. There are abundant germplasm resources of Kam fragrant glutinous rice, mainly of the *japonica* type; rice with different names belonging to the same variety and different varieties with the same names are common. According to the folk classification and modern biological classification of Kam fragrant glutinous rice, Kam fragrant glutinous rice has a high level of genetic diversity, and the folk classification is basically consistent with the biological classification. The folk naming system of rice of the Dong nationality has important reference value for understanding the genetic diversity of Kam fragrant glutinous rice. Kam fragrant glutinous rice is a valuable resource for rice improvement because of its excellent quality and its characteristics of cold and shade tolerance and its abilities to survive in barren environments and resist pests. The germplasm resources of Kam fragrant glutinous rice in the Dong region are still rich in diversity, which is not only related to the local environment but also to the traditional culture of the Dong nationality. It is important to explore the traditional culture related to local rice varieties for the study of agricultural biodiversity.

## 5. Materials and Methods

### 5.1. Study Area

**Figure 7:**
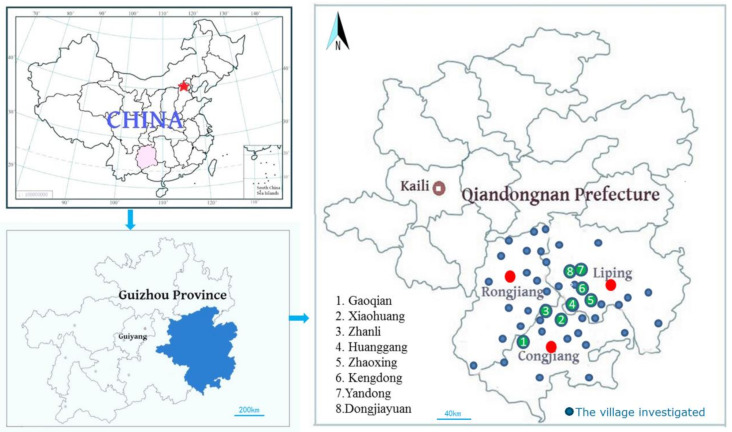
Sketch map of the study area.

The study areas included Liping, Congjiang, and Rongjiang counties (Figure 7), which are located in areas inhabited mainly by the Dong people in the southeast area of Guizhou, China, 108°14′~109°31′ E, 250°16′~260°37′ N. In these three counties, the Dong population is greater than 1.2 million, which is greater than 80% of the total population. This region is mountainous, with a total land area of 2.7 million acres, including 110-thousand acres of rice fields, and is “90% mountains, 0.5% water, and 0.5% field.” The altitude of this region varies from 137 m to 2179 m, with 78.06% forest coverage, and the area is one of eight major forested regions in China. The “rice–fish–duck” system in the Dong communities of Congjiang has been elected as a Globally Important Agricultural Heritage System (GIAHS). This region not only has extremely abundant biodiversity but also possesses rich ethnic traditional cultures and was selected to be a National Ecological Civilization Experimental Zone in 2017.

### 5.2. Collection of Rice Varieties

In 2005, our team began a 12-year collection and research study in the Dong community and the major Kam fragrant glutinous rice-producing areas in the southeast region of Guizhou Province. Through ethnobotanical and cultural anthropological methods, including semi-structured interviews, key informant interviews, and participatory investigation, we examined 42 Dong villages. From every village, 20–30% of farmers were selected as informants. We focused on visiting 500 villagers of ages 18 to 89, with a proportion of males to females of 1:1. Regarding the Kam fragrant glutinous rice varieties, our investigation included the naming and meaning, exchange and origin, management, adaptation to the environment, disease and pest resistance, ethnic traditions and customs, and cultural utilization.

Kam fragrant glutinous rice varieties were collected using three methods: (1) entering the field to investigate the cultivation of Kam fragrant glutinous rice and collecting samples; (2) visiting farmers to investigate and collect information after the fall harvest; and (3) using a group method to collect all Kam fragrant glutinous rice varieties in each village.

### 5.3. Identification of Varieties and Selection of Experimental Materials

The identification of varieties was carried out at three levels: first, the varieties planted by the ordinary farmers were identified; second, the village varieties were identified by the elders, the patriarch, or the leader of a village; and third, representatives composed of elders, patriarchs, and village leaders from 13 villages with large populations and a variety of resources were selected to compare and identify all the germplasm. The representatives from the 13 typical Dong villages, such as Huanggang, Zhanli, Xiaohuang, and Zhaoxing, selected 2 people from each village for a total of 26 people, including 17 males and 9 females, aged from 50 to 83 years old. Finally, those germplasms with different names but of the same variety were merged, different varieties with the same names were separated, and the existing Kam fragrant glutinous rice varieties (including some controversial varieties) were determined according to the folk classification. In this study, all Kam fragrant glutinous rice varieties identified by folk classification and control varieties consisting of two glutinous rice (*japonica*) and two non-glutinous rice (*Indica*) varieties were used as experimental materials, and the seeds of a single panicle of each variety were used as seedling culture materials to prepare for the follow-up work.

### 5.4. Experimental Methods

Seeds from three panicles of each variety were selected and cultured in a greenhouse. When the 3-leaf stage was reached, leaf tissue was harvested, and DNA was extracted using the CTAB method according to Song et al. [44]. The CTAB extraction buffer included 100 mM NaCl, 20 mM EDTA (pH = 8.0), 2% CTAB (*W*/*V*), 100 mM Tris-HCl, and 1.25% SDS; phenol/chloroform/isoamyl alcohol (25:24:1, *V*/*V*); and chloroform/isoamyl alcohol (24:1, *V/V*) were also used.

Primer screening: According to previous studies [18,26]; [34] using SSR markers in local rice varieties from Guizhou, Yunnan, and Guangxi, 40 SSR primers (Supplementary Material Table S1) with a high abundance of polymorphisms were selected for an initial study (to assure the presence of amplified loci in every chromosome). Next, 10 rice varieties with significant morphological differences and different origins were randomly selected for PCR amplification. PCR products were treated and used for vertical gel electrophoresis analysis. The results were observed after silver staining. Twenty SSR primers (Supplementary Material Table S2) that yielded clear, stable bands and polymorphisms were selected (Table 4). After the synthesis of fluorescence-labeled primers, a following batch test was conducted. All primers used in this study were synthesized at The Beijing Genomics Institute (BGI).

PCR amplification: The SSR fluorescence-labeled primer system (20 µL total volume) consisted of ddH_2_O (14.8 µL), dNTPs (0.4 µL), buffer (2 µL), F (0.3 µL of 20 µM primer solution), R (0.3 µL of 20 µM primer solution), DNA template (2 µL), and Taq (0.2 µL). The following amplification program was used: initial denaturation at 94 °C for 5 min; 35 cycles of denaturation at 94 °C for 30 s, annealing at 54 °C for 35 s, and extension at 72 °C for 40 s; and a final extension at 72 °C for 3 min.

Capillary electrophoresis: Formamide and a size standard were mixed at a ratio of 100:1 (*V/V*), and the 9-µL mixture was loaded into the gel. Next, a 10× dilution of 1 µL of PCR product was added. A 3730XL DNA analyzer (Tianjin Genesd Biological Technology Co., LTD, Tianjin, China.) was used for capillary electrophoresis. The fragment (plant) analysis in the Genemarker software was used to analyze the primary data. By comparing the location of the size standard in each lane with the location of the peak in each sample, fragment sizes were obtained.

Data analysis: According to the format requirement by Convert 1.31 software [45], data were recorded in EXCEL and converted into a format required by the POPGENE software by Convert 1.31. POPGENE32 [46] was used for statistical analysis. The NTSYS-pc 2.10e software package was used to calculate the Euclidean distance across experimental samples to construct a phylogenetic tree with 99 samples based on the unweighted pair group method with arithmetic mean (UPGMA) method and to conduct principal coordinate analysis (PCoA).

## Figures and Tables

**Table 1:** Results of the genetic diversity analysis of 95 samples.

Locus	*N*	Chr	*Na*	*H*	*He*	*Ho*	*P*	PIC	Fis	Fit	Fst	*Nm*
RM7	190	3	3	1.0142	0.6173	0.0105	100	0.5363	−0.9973	0.9659	0.9829	0.0043
RM11	188	7	3	0.9564	0.5658	0.0158	100	0.5027	−0.9697	0.9451	0.9725	0.0071
RM21	190	8	6	1.2056	0.6136	0.0105	100	0.5651	−0.9364	0.9657	0.9828	0.0044
RM72	190	5	10	1.8077	0.7963	0.0316	100	0.7685	−0.9561	0.9207	0.9603	0.0103
RM164	190	6	5	0.6321	0.2972	0.0000	100	0.2814	−0.9662	1.0000	1.0000	0.0000
RM190	188	2	6	0.9851	0.5627	0.0000	100	0.4719	−0.9831	1.0000	1.0000	0.0000
RM208	190	11	2	0.6552	0.4625	0.0053	100	0.3556	−0.9754	0.9772	0.9886	0.0029
RM216	190	10	4	1.0237	0.6020	0.0053	100	0.5238	−0.9908	0.9825	0.9913	0.0022
RM219	182	9	7	1.7630	0.8000	0.0105	100	0.7751	−0.9876	0.9742	0.9871	0.0033
RM228	188	10	7	1.0709	0.5044	0.0105	100	0.4742	−0.9853	0.9591	0.9796	0.0052
RM229	190	11	7	1.6659	0.7842	0.0263	100	0.7508	−0.9907	0.9329	0.9664	0.0087
RM235	190	12	4	1.0944	0.6235	0.0053	100	0.5593	−0.9723	0.9831	0.9916	0.0021
RM241	190	4	10	1.5924	0.7278	0.0105	100	0.6866	−0.9856	0.9711	0.9855	0.0037
RM247	188	12	17	2.0606	0.7906	0.0263	100	0.7751	−0.9639	0.9338	0.9669	0.0086
RM254	190	11	5	0.9386	0.4714	0.0105	100	0.4397	−0.9699	0.9553	0.9777	0.0057
RM255	190	4	4	0.6095	0.3202	0.0000	100	0.2902	−0.9897	1.0000	1.0000	0.0000
RM257	190	9	11	1.5132	0.6830	0.0000	100	0.6373	−0.9687	1.0000	1.0000	0.0000
RM263	190	2	4	0.8108	0.4445	0.0000	100	0.3998	−0.9690	1.0000	1.0000	0.0000
RM276	186	6	5	1.3003	0.6990	0.0000	100	0.6413	−0.9823	1.0000	1.0000	0.0000
RM297	190	1	8	1.5844	0.7555	0.0053	100	0.7170	−0.9901	0.9861	0.9930	0.0018
Mean			6.4	1.2142	0.6061	0.0092	100	0.5576	−0.9876	0.9697	0.9849	0.0038

*N =* sample size; Chr = chromosome, *Na* = observed number of alleles; *H =* Shannon’s information index; *He* = gene diversity; *He* = heterozygosity; *P* = polymorphic band ratio; PIC = polymorphic information content; Fis = intra-population self-pollination rate; Fit = inter-population selfing rate; Fst = population differentiation rate; Nm = gene flow.

**Table 2:** Genetic diversity of Kam fragrant glutinous rice in different Dong villiages.

Villiage	*N*	*Na*	*Ne*	*H*	*He*	*Ho*	*AP*	Tot	*P*
Dongjiayuan	14	3.25	2.5100	0.9713	0.5505	0.6843	20	20	100.00
Gaoqian	36	4.2	2.7043	1.0808	0.5677	0.4952	20	20	100.00
Huanggang	50	4.45	2.9852	1.1676	0.6149	0.6874	20	20	100.00
Kengdong	26	3.20	2.1237	0.8353	0.4667	0.4561	20	20	100.00
Xiaohuang	14	2.85	2.2475	0.8370	0.4929	0.3987	18	20	90.00
Yandong	20	3.50	2.6033	1.0157	0.5646	0.4991	20	20	100.00
Zhanli	20	3.65	2.6567	1.0048	0.5420	0.5638	19	20	95.00
Zhaoxing	10	2.45	2.0607	0.7439	0.4626	0.5943	19	20	95.00

*N =* sample size, *Na* = observed number of alleles; *Ne* = effective number of alleles; *H* = Shannon’s information index; *He* = gene diversity; *Ho* = average heterozygosity; PPL = polymorphic band ratio; AP = alleles per polymorphic locus: Tot = total loci; P = proportion of polymorphic loci.

**Table 3:** The genetic distance and similarity of Kam fragrant glutinous rice among different Dong villages.

Village	Dongjiayuan	Gaoqian	Huanggang	Kengdong	Xiaohuang	Yandong	Zhanli	Zhaoxing
Dongjiayuan	****	0.8350	0.8670	0.7822	0.8054	0.8898	0.8468	0.8531
Gaoqian	0.1803	****	0.9197	0.7570	0.9009	0.9602	0.9182	0.8970
Huanggang	0.1428	0.0837	****	0.9111	0.9199	0.9313	0.9196	0.8855
Kengdong	0.2457	0.2784	0.0931	****	0.8390	0.8269	0.8317	0.7854
Xiaohuang	0.2164	0.1043	0.0834	0.1755	****	0.9275	0.8722	0.8738
Yandong	0.1168	0.0406	0.0712	0.1901	0.0752	****	0.9176	0.8734
Zhanli	0.1663	0.0854	0.0838	0.1843	0.1367	0.0860	****	0.9046
Zhaoxing	0.1589	0.1087	0.1216	0.2415	0.1349	0.1353	0.1003	****

The genetic similarity is above the asterisk line (****), and the genetic distance is the lower side of the asterisk line.

**Table 4:** Results of SSR primer screening.

Chromosome	Locus	Primer Forward	Primer Reverse
1	RM297	5′-TCTTTGGAGGCGAGCTGAG-3′	5′-CGAAGGGTACATCTGCTTAG-3′
2	RM208	5′-TCTGCAAGCCTTGTCTGATG-3′	5′-TAAGTCGATCATTGTGTGGACC-3′
2	RM263	5′-CCCAGGCTAGCTCATGAACC-3′	5′-GCTACGTTTGAGCTACCACG-3′
3	RM7	5′-TTCGCCATGAAGTCTCTCG-3′	5′-CCTCCCATCATTTCGTTGTT-3′
4	RM241	5′-GAGCCAAATAAGATCGCTGA-3′	5′-TGCAAGCAGCAGATTTAGTG-3′
4	RM255	5′-TGTTGCGTGTGGAGATGTG-3′	5′-CGAAACCGCTCAGTTCAAC-3′
5	RM165	5′-TCTTGCCCGTCACTGCAGATATCC-3′	5′-GCAGCCCTAATGCTACAATTCTTC-3′
6	RM190	5′-CTTTGTCTATCTCAAGACAC-3′	5′-TTGCAGATGTTCTTCCTGATG-3′
6	RM276	5′-CTCAACGTTGACACCTCGTG-3′	5′-TCCTCCATCGAGCAGTATCA-3′
7	RM11	5′-TCTCCTCTTCCCCCGATC-3′	5′-ATAGCGGGCGAGGCTTAG-3′
8	RM72	5′-CCGGCGATAAAACAATGAG-3′	5′-GCATCGGTCCTAACTAAGGG-3′
9	RM219	5′-CGTCGGATGATGTAAAGCCT-3′	5′-CATATCGGCATTCGCCTG-3′
9	RM257	5′-CAGTTCCGAGCAAGAGTACTC-3′	5′-GGATCGGACGTGGCATATG-3′
10	RM216	5′-CATAGTGGAGTATGCAGCTGC-3′	5′-CCTTCTCCCAGTCGTATCTG-3′
10	RM228	5′-CTGGCCATTAGTCCTTGG-3′	5′-GCTTGCGGCTCTGCTTAC-3′
11	RM21	5′-ACAGTATTCCGTAGGCACGG-3′	5′-CTCCATGAGGGTGGTAGAG-3′
11	RM229	5′-CACTCACACGAACGACTGAC-3′	5′-CGCAGGTTCTTGTGAAATGT-3′
11	RM254	5′-AGCCCCGAATAAATCCACCT-3′	5′-CTGGAGGAGCATTTGGTAGC-3′
12	RM19	5′-CAAAAACAGAGCAGATGAC-3′	5′-CTCAAGATGGACGCCAAGA-3′
12	RM247	5′-TAGTGCCGATCGATGTAACG-3′	5′-CATATGGTTTTGACAAAGCG-3′

## Data Availability

Data is contained within the article or Supplementary Material.

## Supplementary Materials

The following are available online at https://www.mdpi.com/article/10.3390/plants10091898/s1, Additional file: Table S1. Variety code and Dong ethonic name of Kam fragrant Glutinous rice in this study; Table S2. Alternative 40 pairs of primer information in this study.docx.

## Abbreviations

BGI: Beijing Genomics Institute; CTAB: Cetyltrimethylammonium Bromide; DNA: Deoxyribo Nucleic Acid; dNTPs: Deoxyribonucleoside Rriphosphates; EDTA: Ethylene Diamine Tetraacetic Acid; GIAHS: Globally Important Agricultural Heritage Systems; PCoA: Principal coordinate analysis; PCR: Polymerase chain reaction; PPL: Percentage of polymorphic bands; SDS; Sodium dodecyl sulfate; SSR: Simple sequence repeat; UPGMA: Unweighted pair group method with arithmetic mean.
