# Supplementary material for: Genetic Diversity Evaluation and Conservation of Kam Fragrant Glutinous Rice (Oryza sativa L.) Germplasm in Southeast Guizhou, China
Source: Plants (Basel). 2021 Sep 14;10(9):1898. doi: 10.3390/plants10091898 (PMC8472733; doi:10.3390/plants10091898)
Supplement: Supplementary file 1 [file plants-10-01898-s001.zip › Additional file 1 Table S2..pdf]

Additional file 1

Table S2. Variety code and Dong ethonic name of Kam fragrant glutinous rice in this study.

Code	Dong name	Code	Dong name	Code	Dong name
GQ1-1	Kgoux maoh	KD9-34	Kgoux lieeh laox	DJ7-67	Kgoux ral jix daeml
ZX1-2	Kgoux jol laox	ZL3-35	Kgoux lieev longl	HG17-68	Kgoux yangc longl
DJ1-3	Kgoux bedh jul ral	XH4-36	Kgoux jinc badh	ZX5-69	Kgoux jav raih
KD1-4	Kgoux yongp dongx	HG5-37	Kgoux guanl	DJ4-70	Kgoux shol deec
HG1-5	Kgoux naemx nimh pangp	XH6-38	Kgoux bieeh	DJ5-71	Kgoux bedh jul naeml
ZL1-6	Kgoux gkogh ral	HG8-39	Kgoux bieengh mant	DJ6-72	Kgoux div bingc naeml
HG2-7	Kgoux padh nganx	YD4-40	Kgoux junl zinc	ZL7-73	Kgoux badh naemv
HG3-8	Kgoux Gaos Sinp	KD10-41	Kgoux gongh genx	YD8-74	Kgoux meec guanl
DJ2-9	Kgoux bic ral	XH7-42	Kgoux touv munv	KD11-75	Kgoux baix tangc
DJ3-10	Kgoux padh nyox	GQ5-43	Kgoux gongp guih laox	GQ12-76	Kgoux dongc lac
KD2-11	Kgoux joh naemv	ZL4-44	Kgoux dal naeml	ZL8-77	Kgoux sadh manh
HG4-12	Kgoux bic bagh	HG9-45	Kgoux banl jinc	HG18-78	Kgoux jul
XH1-13	Kgoux lieec laox jal raih	HG10-46	kgoux gkaox dah laox	XH9-79	Kgoux gongh gunl
KD3-14	Kgoux bienl gueec laox	GQ6-47	Kgoux longv xaox	ZL9-80	Kgoux yangc
KD4-15	Kgoux naeml jal raih	GQ7-48	Kgoux gunv	ZL10-81	Kgoux juh xil maenv
XH2-16	Kgoux naeml ragx	GQ8-49	Kgoux gkunl ral	GQ13-82	Kgoux siv wangc
ZX2-17	Kgoux ral jol ral laox	HG11-50	Kgoux bic bagx	GQ14-83	Kgoux yongc laox
YD1-18	Kgoux binl guic laox	HG12-51	Kgoux tidl xil maenv	GQ15-84	Kgoux dangl
YD2-19	Kgoux bieenx longc touc	GQ9-52	Kgoux bagx liix	HG19-85	Kgoux yongc
XH3-20	Kgoux zinc	ZX3-53	Kgoux jal ral	KD12-86	Kgoux bongh beic
YD10-21	Kgoux jeemh	GQ10-54	Kgoux daov ral	HG20-87	Kgoux bedh gueenx
HG5-22	Kgoux jinc dongl	YD5-55	Kgoux lieec zhul	HG21-88	Kgoux nunx raih
GQ2-23	Kgoux daov	YD6-56	Kgoux yangc	YD9-89	Kgoux meev jal
KD5-24	Kgoux bieec gunl	YD7-57	Kgoux ginh	HG22-90	Kgoux weenx ninl
GQ3-25	Kgoux qaenp	GQ11-58	Kgoux longv bieec	HG23-91	Kgoux logc xil maenv
HG6-26	Kgoux dangh dal	HG8-59	Kgoux tunc	KD13-92	Kgoux jeec
KD6-27	Kgoux bagx sal	HG13-60	Kgoux yeec	GQ16-93	Kgoux gaoh jiml
ZL2-28	Kgoux lieec jul	HG14-61	Kgoux lieel zhul lagx	GQ17-94	Kgoux longv daiv
KD7-29	Kgoux beenh weenx jal	HG15-62	Kgoux div gnov	GQ18-95	Kgoux gouc
YD3-30	Kgoux bagx dangp	HG16-63	Kgoux deeh ngox	**XZ1-96**	Bai mao dao
GQ4-31	Kgoux gongp guih lagx	ZL5-64	Kgoux yangc dangh	**ZY1-97**	Zhu shi nuo
KD8-32	Kgoux diol deex mangc	ZL6-65	Kgoux wangl niic	**XY1-98**	Ma nuo
HG7-33	Kgoux bienl gueec lagx	ZX4-66	Kgoux bic ral	**CH1-99**	Xiao huang ke zhan

NOTE: (1) GQ1-1：Indicates that the variety is the first sample of Gaoqian vellige, corresponding to the number 1 of the picture, and that "HG" is the abbreviation of "Huanggang vellige", and so on; (2) Contrast variety：*Japonica*（XZ1-96，ZY1-97）, *Indica(*XY1-98, CH1-99), ZY1-97 and XY1-98 are glutinous, XZ1-96 and CH1-99 are no-glutinous.
