# Supplementary material for: Genetic Diversity Evaluation and Conservation of Kam Fragrant Glutinous Rice (Oryza sativa L.) Germplasm in Southeast Guizhou, China
Source: Plants (Basel). 2021 Sep 14;10(9):1898. doi: 10.3390/plants10091898 (PMC8472733; doi:10.3390/plants10091898)
Supplement: Supplementary file 1 [file plants-10-01898-s001.zip › Additional file 1 Table S1..pdf]

Table S1. Alternative 40 pairs of primer information in this study.

Chromosome	Locus	Primer forward	Primer reverse
1	RM5	TGCAACTTCTAGCTGCTCGA	GCATCCGATCTTGATGGG
1	RM24	GAAGTGTGATCACTGTAACC	TACAGTGGACGGCGAAGTCG
1	RM246	GAGCTCCATCAGCCATTCAG	CTGAGTGCTGCTGCGACT
1	RM297	TCTTTGGAGGCGAGCTGAG	CGAAGGGTACATCTGCTTAG
2	RM208	TCTGCAAGCCTTGTCTGATG	TAAGTCGATCATTGTGTGGACC
2	RM262	CATTCCGTCTCGGCTCAACT	CAGAGCAAGGTGGCTTGC
2	RM263	CCCAGGCTAGCTCATGAACC	GCTACGTTTGAGCTACCACG
3	RM143	GTCCCGAACCCTAGCCCGAGGG	AGAGGCCCTCCACATGGCGACC
3	RM218	TGGTCAAACCAAGGTCCTTC	GACATACATTCTACCCCCGG
3	RM282	CTGTGTCGAAAGGCTGCAC	CAGTCCTGTGTTGCAGCAAG
4	RM273	GAAGCCGTCGTGAAGTTACC	GTTTCCTACCTGATCGCGAC
4	RM255	TGTTGCGTGTGGAGATGTG	CGAAACCGCTCAGTTCAAC
4	RM241	GAGCCAAATAAGATCGCTGA	TGCAAGCAGCAGATTTAGTG
5	RM13	TCCAACATGGCAAGAGAGAG	GGTGGCATTCGATTCCAG
5	RM164	TCTTGCCCGTCACTGCAGATATCC	GCAGCCCTAATGCTACAATTCTCC
5	RM249	GGCGTAAAGGTTTTGCATGT	ATGATGCCATGAAGGTCAGC
6	RM190	CTTTGTCTATCTCAAGACAC	TTGCAGATGTTCTTCCTGATG
6	RM204	GTGACTGACTTGGTCATAGGG	GCTAGCCATGCTCTCGTACC
6	RM276	CTCAACGTTGACACCTCGTG	TCCTCCATCGAGCAGTATCA
7	RM11	TCTCCTCTTCCCCCGATC	ATAGCGGGCGAGGCTTAG
7	RM18	TTCCCTCTCATGAGCTCCAT	GAGTGCCTGGCGCTGTAC
7	RM180	CTACATCGGCTTAGGTGTAGCAACACG	ACTTGCTCTACTTGTGGTGAGGGACTG
7	RM192	GCGGCGGATCATGAATTGCGAG	CTTGTTCCCCGGCGTCGAGTCC
8	RM32	AGTCTACGTGGTGTACACGTGG	AAGTCCATTACTCTCCTCCC
8	RM44	ACGGGCAATCCGAACAACC	TCGGGAAAACCTACCCTACC
8	RM152	GAAACCACCACACCTCACCG	CCGTAGACCTTCTTGAAGTAG
9	RM219	CGTCGGATGATGTAAAGCCT	CATATCGGCATTCGCCTG
9	RM242	GGCCAACGTGTGTATGTCTC	TATATGCCAAGACGGATGGG
9	RM296	CACATGGCACCAACCTCC	GCCAAGTCATTCACTACTCTGG
9	RM257	CAGTTCCGAGCAAGAGTACTC	GGATCGGACGTGGCATATG
10	RM216	GCATGGCCGATGGTAAAG	TGTATAAAACCACACGGCCA
10	RM228	CTGGCCATTAGTCCTTGG	GCTTGCGGCTCTGCTTAC
10	RM258	TGCTGTATGTAGCTCGCACC	TGGCCTTTAAAGCTGTCGC
11	RM21	ACAGTATTCCGTAGGCACGG	CTCCATGAGGGTGGTAGAG
11	RM206	CCCATGCGTTTAACTATTCT	CGTTCCATCGATCCGTATGG
11	RM229	CACTCACACGAACGACTGAC	CGCAGGTTCTTGTGAAATGT
11	RM254	AGCCCCGAATAAATCCACCT	CTGGAGGAGCATTTGGTAGC

Chromosome	Locus	Primer forward	Primer reverse
12	RM19	CAAAAACAGAGCAGATGAC	CTCAAGATGGACGCCAAGA
12	RM235	AGAAGCTAGGGCTAACGAAC	TCACCTGGTCAGCCTCTTTC
12	RM247	TAGTGCCGATCGATGTAACG	CATATGGTTTTGACAAAGCG
3	RM135	CTCTGTCTCCTCCCCCGCGTCG	TCAGCTTCTGGCCGGCCTCCTC
3	RM426	ATGAGATGAGTTCAAGGCCC	AACTCTGTACCTCCATCGCC
8	RM72	CCGGCGATAAAACAATGAG	GCATCGGTCCTAACTAAGGG
8	RM284	ATCTCTGATACTCCATCCATCC	CCTGTACGTTGATCCGAAGC
3	RM36	CAACTATGCACCATTGTCGC	GTACTCCACAAGACCGTACC
3	RM7	TTCGCCATGAAGTCTCTCG	CCTCCCATCATTTCGTTGTT
3	RM22	GGTTTGGGAGCCCATAATCT	CTGGGCTTCTTTCACTCGTC
8	RM25	GGAAAGAATGATCTTTTCATGG	CTACCATCAAAACCAATGTTC
8	RM80	TTGAAGGCGCTGAAGGAG	CATCAACCTCGTCTTCACCG
8	RM137	GACATCGCCACCAGCCCACCAC	CGGGTGGTCCCCGAGGATCTTG
